# Genome-Scale Discovery of DNA-Methylation Biomarkers for Blood-Based Detection of Colorectal Cancer

**DOI:** 10.1371/journal.pone.0050266

**Published:** 2012-11-28

**Authors:** Christopher P. E. Lange, Mihaela Campan, Toshinori Hinoue, Roderick F. Schmitz, Andrea E. van der Meulen-de Jong, Hilde Slingerland, Peter J. M. J. Kok, Cornelis M. van Dijk, Daniel J. Weisenberger, Hui Shen, Robertus A. E. M. Tollenaar, Peter W. Laird

**Affiliations:** 1 Department of Surgery, Groene Hart Hospital, Gouda, The Netherlands; 2 Department of Clinical Chemistry, Groene Hart Hospital, Gouda, The Netherlands; 3 Department of Pathology, Groene Hart Hospital, Gouda, The Netherlands; 4 Department of Surgery, Leiden University Medical Center, Leiden, The Netherlands; 5 Department of Gastroenterology and Hepatology, Leiden University Medical Center, Leiden, The Netherlands; 6 Department of Surgery, University of Southern California, Keck School of Medicine, USC/Norris Comprehensive Cancer Center, Los Angeles, California, United States of America; 7 Department of Biochemistry and Molecular Biology, University of Southern California, Keck School of Medicine, USC/Norris Comprehensive Cancer Center, Los Angeles, California, United States of America; 8 USC Epigenome Center, University of Southern California, Los Angeles, California, United States of America; Dartmouth Medical School, United States of America

## Abstract

**Background:**

There is an increasing demand for accurate biomarkers for early non-invasive colorectal cancer detection. We employed a genome-scale marker discovery method to identify and verify candidate DNA methylation biomarkers for blood-based detection of colorectal cancer.

**Methodology/Principal Findings:**

We used DNA methylation data from 711 colorectal tumors, 53 matched adjacent-normal colonic tissue samples, 286 healthy blood samples and 4,201 tumor samples of 15 different cancer types. DNA methylation data were generated by the Illumina Infinium HumanMethylation27 and the HumanMethylation450 platforms, which determine the methylation status of 27,578 and 482,421 CpG sites respectively. We first performed a multistep marker selection to identify candidate markers with high methylation across all colorectal tumors while harboring low methylation in healthy samples and other cancer types. We then used pre-therapeutic plasma and serum samples from 107 colorectal cancer patients and 98 controls without colorectal cancer, confirmed by colonoscopy, to verify candidate markers. We selected two markers for further evaluation: methylated *THBD* (THBD-M) and methylated *C9orf50* (C9orf50-M). When tested on clinical plasma and serum samples these markers outperformed carcinoembryonic antigen (CEA) serum measurement and resulted in a high sensitive and specific test performance for early colorectal cancer detection.

**Conclusions/Significance:**

Our systematic marker discovery and verification study for blood-based DNA methylation markers resulted in two novel colorectal cancer biomarkers, THBD-M and C9orf50-M. THBD-M in particular showed promising performance in clinical samples, justifying its further optimization and clinical testing.

## Introduction

Colorectal cancer (CRC) is a common disease with an estimated 143,460 new cases in the USA in 2012 [1]. CRC is the third most frequently diagnosed cancer in males and females in the Western world and a significant percentage of patients who present with CRC will have distant metastases (stage IV) at diagnosis, which is often incurable. It is clear that localized cancer (stage I/II) detected early is more amenable to curative therapy, offering superior prognosis [2], [3]. Accordingly, diagnostic methods that result in early detection of malignant or even premalignant disease could have considerable clinical benefits, reducing mortality and morbidity of patients with colorectal cancer. Available potential screening techniques for CRC include fecal occult blood test, double contrast barium enema, endoscopy, with preference for pancolonoscopy, and virtual colonoscopy [4], [5]. The measurement of serum carcinoembryonic antigen (CEA) has also been suggested as a possible screening modality but it lacks sufficient sensitivity to detect CRC at an early stage, and its level is also elevated in non-malignant diseases (e.g. diverticulitis, gastritis, diabetes) [6].

An optimal screening test is expected to be highly sensitive and specific, pose no risk to the patients, and have high patient acceptance. It should also be cost effective and easy to perform. As current screening procedures lack sufficient positive predictive value, require unpleasant preparation or cause discomfort, there is a need to develop new non-invasive tests for the detection of CRC at a stage early enough for treatment to be successful. DNA methylation markers are promising tools that could be useful for early cancer detection. In the past decade it has become clear that cancer cells have aberrant patterns of DNA methylation and that these abnormalities can be detected in tumor-derived DNA found in the plasma or serum of cancer patients [7], [8]. Several studies have documented the presence of free DNA derived from solid tumors in the bloodstream of cancer patients, which raises the possibility of detecting these cancer-specific molecules in subjects with existing disease [9]–[22].

A number of studies have already reported the use of DNA methylation markers for blood-based detection of CRC with varying results [9]–[22]. However, most of these studies have relied on testing a limited number of pre-selected genes and on the use of non-quantitative detection methods, such as gel-based methylation-specific PCR. The aim of this study is to identify blood-based DNA methylation biomarkers for CRC using a genome-scale DNA methylation approach for marker discovery and to test the selected markers in pre-operative clinical blood specimens from patients undergoing curative surgery for CRC.

## Methods

### Human Samples and Ethics Statement

The local and regional institutional review boards approved this study. Informed consent was obtained from all participating patients and controls. Pre-therapeutic plasma and serum samples were obtained from CRC patients in the outpatient clinic via phlebotomy of the median cubital vein from April 2008 to December 2011. Plasma and serum were isolated within 30 minutes of venapuncture as previously described [22]. Each plasma or serum sample was further divided into two separate tubes and stored at −80°C until processing. The serum CEA was measured at each venapuncture in CRC patients.

Controls were defined as subjects without CRC or any malignancy in the past five years and were included in this study at the endoscopy department. Individuals undergoing colonoscopy, who showed no sign of a colorectal malignancy, were eligible to participate. Indications for colonoscopy for these patients were surveillance colonoscopies because of inflammatory bowel disease (IBD; Crohn's disease or Ulcerative Colitis), positive family history of CRC, gastro-intestinal complaints or rectal blood loss. An experienced gastroenterologist performed all colonoscopies. Patients with mild, controlled IBD were included as long as it was possible to reliably inspect the colonic mucosa at colonoscopy *and* if the surveillance biopsies that are routinely taken along the whole colorectal tract were pathologically normal (showing no signs of dysplasia). Plasma and serum samples were isolated from these individuals using the same protocol as for the CRC patients.

CRC tissue was obtained during the surgical resection of the tumor and immediately sent to the pathologist. The pathologist dissected a representative part of the tumor and stored the fresh-frozen sample at −80°C within one hour after surgical resection. In addition, a pathologically normal colon sample was taken at least 10 cm away from the edge of the tumor and stored in the same way.

### Marker Discovery: Technologies and Datasets

In the marker discovery phase of this study we used DNA methylation data generated by Illumina Infinium HumanMethylation27 BeadChip® (HM27) and the HumanMethylation450 BeadChip® (HM450) platforms. The Infinium assay quantifies DNA methylation levels at specific cytosine residues adjacent to guanine residues (CpG loci), by calculating the ratio (β-value) of intensities between locus-specific methylated and unmethylated bead-bound probes. The β-value is a continuous variable, ranging from 0 (unmethylated) to 1 (fully methylated) [23]. The HM27 BeadChip® assesses the DNA methylation level of 27,578 CpG sites located at the promoter regions of 14,495 protein-coding genes. The HM450 BeadChip® evaluates DNA methylation status of 482,421 CpG loci and covers 99% of RefSeq genes and 96% of UCSC CpG islands (www.ncbi.nlm.nih.gov/RefSeq, www.illumina.com).

We used available Infinium HM27 and HM450 data from 711 colorectal tumors, 53 matched adjacent-normal colonic tissue samples and 10 peripheral blood lymphocyte (PBL) samples of healthy individuals to identify and verify candidate DNA methylation tumor markers. In addition, we used Infinium data from publicly available data sets (GEO and TCGA) representing 274 healthy PBL samples and 4,201 malignant tissue specimens from 15 different cancer types to maximize CRC specificity (see Table 1). The β-values of 611 CRC tumors and 24 matched adjacent-normal colonic tissue samples were retrieved from the DNA methylation dataset for CRCs posted on The Cancer Genome Atlas (TCGA) Data Portal (http://tcga-data.nci.nih.gov/tcga/tcgaHome2.jsp). Data of the other 100 CRC tumors and 29 matched adjacent-normal colonic tissue samples were generated at the USC Epigenome Center in a previous study [24]. Infinium data of 274 PBL samples were downloaded from the Gene Expression Omnibus (GEO) database at the National Center for Biotechnology Information (NCBI, http://www.ncbi.nlm.nih.gov/geo/, accession number GSE 19711). The data for the 10 remaining PBL samples were generated using the HM27 platform for a previous marker discovery study at the USC Epigenome Center [25]. Document S1 summarizes the archive numbers of all publicly available GEO and TCGA datasets used in this study. DNA methylation status was assessed using the HM27 BeadChip for 336 CRC samples, 29 normal colon samples and 274 PBL samples (Table 1). For the remaining 375 CRC tumors and 24 normal colon samples, the DNA methylation status was evaluated with the HM450 BeadChip (Table 1). We also generated HM450 data on two PBL samples from the collection of 10 studied on the HM27 platform. Of the 375 CRC, data from only 40 samples were available at the time we performed the marker discovery. DNA methylation data of the other 335 CRC tumors were not used in the marker selection algorithm. We did, however, use these data to verify the final two markers selected.

**Table 1:** Overview of samples and data sets used for biomarker discovery.

	DNA Methylation Analysis Platform	
Sample collections (TCGA abbr)	HM27	HM450
GEO Colorectal cancer tumor	100	
GEO PBL from healthy controls	10	2**
GEO Normal colorectal tissue*	29	
GEO PBL from healthy controls	274	
TCGA Normal colorectal tissue (COAD/READ)		24
TCGA CRC tumor discovery set (COAD/READ)	236	40
TCGA CRC tumor verification set (COAD/READ)		335
TCGA Acute myeloid leukemia (LAML)	192	192**
TCGA Bladder urothlial carcinoma (BLCA)		78
TCGA Breast invasive carcinoma (BRCA)	316	498
TCGA Gastric adenocarcinoma (STAD)	82	70
TCGA Glioblastoma mulitforme (GBM)	296	
TCGA Skin Cutaneous Melanoma (SKCM)		241
TCGA Lung adenocarcinoma (LUAD)	128	222
TCGA Lung squamous cell carcinoma (LUSC)	134	150
TCGA Ovarium serous adenocarcinoma (OV)	405	
TCGA Pancreas (PAAD)		30
TCGA Prostate (PRAD)	154	
TCGA Renal clear cell (KIRC)	219	283
TCGA Thyroid carcinoma (THCA)		230
TCGA Head and Neck squamous cell carcinoma (HNSC)		292
TCGA Uterine corpus endometrioid carcinoma (UCEC)	117	256

* normal samples were obtained from surgical specimens of CRC patients, at least 10 cm from the tumor margins.

** these samples were among the samples run on the HM27 platform.

### Marker Discovery: Filter Criteria

**Figure 1:**
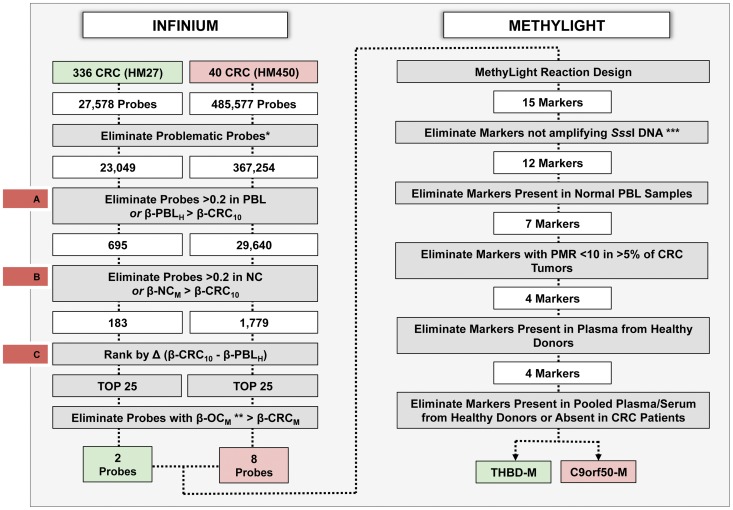
Schematic representation of colorectal cancer marker discovery and verification pipeline. We used DNA methylation data from the Infinium HumanMethylation27 Beadchip (HM27) and HumanMethylation450 Beadchip (HM450) Infinium platforms to screen 27,578 (HM27) and 482,421 (HM450) CpG loci for their methylation status in CRC samples, PBL samples from healthy subjects, paired normal colorectal tissue samples (NC) and 15 other types of cancer (OC). We used a stepwise approach eliminating probes that failed in any of the samples, probes that contained SNPs or repeat sequences, probes with a highest PBL β-value (β-PBL_H_) or a mean normal colon tissue β-value (β-NC_M_) higher than the associated 10th percentile of CRC tumor β-values (β-CRC_10_) or higher than 0.2 in any of the PBL or NC samples (Infinium panel). The remaining probes were ranked based on the difference between β-CRC_10_ and β-PBL_H_ and the top 25 were selected from both datasets (HM27 and HM450) for filtering against OC samples. Probes with a mean OC β-value higher than the associated mean CRC β-value (β-CRC_M_) were eliminated. A total of 15 MethyLight reactions (markers) were designed for 10 probes and tested in a sequence of verification steps (MethyLight panel). Markers were eliminated if their performance was suboptimal in controls such as *in vitro* methylated *Sss*1 DNA, PBL and plasma samples from healthy controls and CRC tumor tissues. Markers were also eliminated if they failed to detect CRC methylated DNA in pooled plasma and serum from CRC patients. Two markers met all the selection criteria and were advanced in the pipeline for further verification on individual patient samples. (*Probes that failed in any of the samples, as well as those that included SNPs and repeat sequences; **Other cancer types used in this study are summarized in Table 1, ***M.*Sss*I treated DNA).

**Figure 2:**
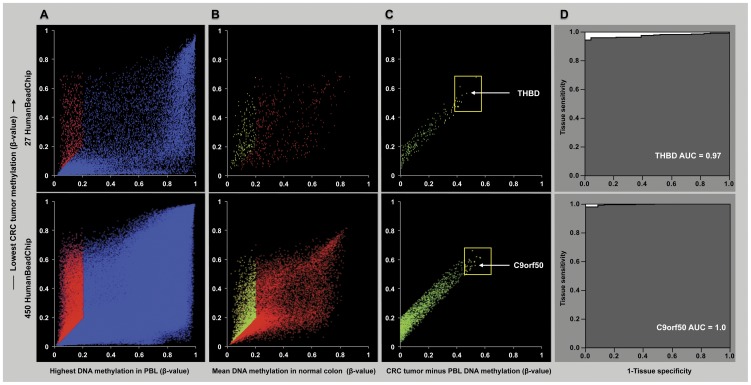
Scatterplot representation of marker discovery process and ROC curves. A (top figure: HM27, bottom figure: HM450), scatterplots of the highest PBL β-value (β-PBL_H_) of 10 (HM27) and 2 (HM450) healthy control samples (X-axis) against the associated 10th percentile of CRC tumor β-values (β-CRC_10_) on the Y-axis. The blue dots represent the eliminated probes (HM27: n = 23,049; HM450: n = 367,833) and the red dots (HM27: n = 695; HM450: n = 30,207) represent the retained probes with a β-CRC_10_>β-PBL_H_ or a β-PBL_H_<0.2. B, scatterplots of the mean normal colon tissue β-value (β-NC_M_) for the retained probes from Panel A (X-axis) against the associated β-CRC_10_ (Y-axis). The red dots (HM27: n = 512; HM450: n = 28,428) represent the eliminated probes, the green dots represent the retained probes (HM27: n = 183; HM450: n = 1779) with a β-CRC_10_>β-NC_M_ or a β-NC_M_<0.2. C, scatterplots of the retained probes from Panel B (green) displayed by the difference between β-CRC_10_ and β-PBL_H_ (X-axis) against the associated β-CRC_10_ (Y-axis). The dots within the yellow square are the probes selected for additional filtering against other types of cancer. The white arrows point out the probes of the two candidate markers. D, ROC curves for the probes used in the multiplex reaction based on methylation β-values of 335 independent colorectal cancer samples and 23 independent matched normal colorectal tissue samples (the DNA methylation data of these samples were not used in the marker discovery pipeline). The dark grey color is the area under the curve.

We employed a multistep filtering process in the discovery phase of this study. DNA methylation data generated by the two different BeadChips (HM27 and HM450) were analyzed separately, but using the same filtering steps (Figure 1 and 2). We started by excluding all Infinium probes that failed in any of the samples. HM27 probes that were not uniquely aligned to the human genome (hg19, GRCh37), or that were associated with single nucleotide polymorphisms (SNPs) within 10 basepairs of the target CpG (identified using the NCBI dbSNP builds 126 and 128), or probes that covered repetitive elements (identified by RepeatMasker) were also excluded. We determined the highest β-value for each probe in 10 healthy PBL samples (β-PBL_H_) and the 10^th^ percentile of the CRC tumor β-values (β-CRC_10_) (Figure 2A). We excluded all probes with a β-PBL_H_ higher than 0.2 or higher than the associated β-CRC_10_. We then filtered the remaining probes against normal colon tissue and 15 other cancer types. In detail, we determined the mean β-value of each probe in normal colon tissue (β-NC_M_) and eliminated all probes that had a β-NC_M_ value higher than 0.2 or higher than the associated β-CRC_10_ value (Figure 2B). We selected the top 25 markers in both datasets after ranking the probes based on the difference between β-CRC_10_ and β-PBL_H_ (Figure 2C). For the filtering against other cancer types, we determined the mean β-value (β-OC_M_) for the remaining probes in each cancer type and eliminated all probes that had a β-OC_M_ higher than the mean CRC β-value (β-CRC_M_). The remaining probes were selected for MethyLight reaction design and further evaluation.

### DNA Extraction and Bisulfite Modification

DNA from two healthy PBL samples and 25 CRC tumor samples were extracted according to the previously described protocol [25]. DNA from plasma and serum samples was extracted using the QIAamp® Circulating Nucleic Acid Kit (Qiagen), specially designed to recover a maximum amount of circulating cell-free DNA from serum or blood. The Zymo® EZ DNA methylation kit (Zymo Research) was used to bisulfite convert the extracted DNA. All extractions and conversions were performed according to the manufacture's instructions. The quality and quantity of the bisulfite-converted DNA, as well as the completeness of the bisulfite conversion, were assessed using a panel of quality control reactions as previously described [26].

### MethyLight Analysis

The MethyLight assay was performed as previously described [27]. The sequence of the MethyLight primers and probes used in these analyses are described in Table S1. The MethyLight reactions were evaluated in four steps. First, M.*Sss*I (New England Biolabs) treated PBL DNA (Promega) was used to determine if the reaction amplified *in vitro* methylated control DNA [28]. Reactions with a cycle threshold [C(t)] higher than 35 were excluded. Secondly, the reactions were screened against 50 ng PBL DNA from two healthy individuals. Reactions with C(t) values lower than 40 were excluded. The remaining reactions were tested on 25 CRC DNA samples, using an ALU-based MethyLight reaction and an M.*Sss*1 DNA standard curve to calculate the Percentage of Methylated Reference (PMR) as described previously [27], [29]. Reactions with a PMR<10 in more than one CRC tumors were eliminated. Finally, the reactions were tested in 10 plasma samples from healthy donors (equivalent of 100 µl plasma) and ranked according to their C(t) values. Reactions with C(t) values less than 50 in one or more of these samples were eliminated.

### Digital MethyLight Analysis

#### Pooled clinical samples

Digital MethyLight is a quantitative PCR technique in which bisulfite-converted DNA is analyzed using the MethyLight PCR assay in a distributive fashion over 96 reaction chambers for each sample. This technique is an efficient and effective method of obtaining DNA methylation information for samples with small amounts of DNA and was performed as described earlier [25], [30]. We prepared four separate pools of plasma and four pools of serum DNA to test candidate markers (Pool 1 = 16 controls (without IBD), Pool 2 = 16 patients with mild IBD, Pool 3 = 16 patients with stages I/II CRC and Pool 4 = 16 patients with stages III/IV CRC). Each of these pools contained DNA from 190 µl of plasma or serum from each of the 16 individuals in the pool. For each reaction we first tested DNA from 50 µl plasma or serum of each pool. For the reactions that did not result in any PCR amplifications (hits) with 50 µl, the volume was increased to a 150 µl equivalent. Finally, reactions that did not result in any hits in the CRC pools or gave hits in the controls with or without IBD were excluded.

The two candidate markers that survived this elimination process were labeled with different fluorophores. This enabled reaction specific colored PCR outcomes that allowed us to distinguish hits from each of these markers when they were run together (multiplex). All probes and primers were synthesized by Biosearch Technology, Inc, Novota, California, USA.

#### Individual clinical samples

The two-marker multiplex was tested on plasma and serum samples from 75 independent CRC patients and 70 controls with a test volume of 1 ml. Table 2 gives an overview of the clinical characteristics of the CRC patients and control subjects used for clinical marker testing in this study.

**Table 2:** Clinical characteristics of CRC patients and controls used for plasma and serum analysis.

	CONTROLS				CRC PATIENTS						
Sample set	Samples	Median Age in Year (Range)	Female/Male	Crohn's disease/Ulcerative colitis	Samples	Median Age in Year (Range)	Female/Male	Rectum n (%)	Colon n (%)	Stage	n (%)
	n				n						
Pooled set	32	59 (40–85)	16/16	7/9	31	63 (41–84)	15/16	5 (16)	26 (84)	I	9 (31)
										II	6 (19)
										III	14 (45)
										IV	2 (1)
Independent set	66	61 (39–85)	22/48	2/8	75	72 (51–92)	34/41	19 (25)	56 (75)	I	19 (25)
										II	24 (32)
										III	31 (41)
										IV	1 (0)

### Statistical Analysis

The computation of confidence intervals of areas under the curve (AUCs) and the statistical tests were conducted in R (version 2.14.0), with the R package pROC [31]. The method as proposed by DeLong and colleagues [32] was used for the test.

## Results

### Marker Discovery in Genome-Scale DNA Methylation Data Sets

We performed a stepwise marker discovery analysis using available DNA methylation data sets from a large number of CRC tumors, 15 different other cancer types, and control samples from plasma, PBL and matched adjacent-normal colonic tissues (Figures 1 and 2, Table 1) to identify CRC DNA methylation markers. We used data generated using two different Illumina Infinium HumanMethylation BeadChip platforms, HM27 and HM450 (see Methods section). After removing potentially problematic probes, probe sequences that overlapped SNPs or repetitive elements, and probes that failed to perform in all samples, there were 23,049 HM27 probes and 367,254 HM450 probes. Of these probes, 695 remained in the HM27 group and 29,640 in the HM450 group, after eliminating those that had higher DNA methylation levels in the healthy PBL samples than in CRC tumors. In addition, we excluded all probes with higher DNA methylation in normal colon tissue than in CRC and ranked the remaining probes based on the difference between healthy PBL and CRC tumor DNA methylation. The DNA methylation status of the combined top 50 probes (ranked by the greatest difference between healthy PBL and CRC DNA methylation) were compared between CRC and 15 other types of cancer. Probes with a higher mean DNA methylation level in any other type of cancer than in CRC were excluded. Ten CRC-specific candidate probes (associated with ten unique gene promoters) remained, which showed higher mean DNA methylation values in CRC than the mean corresponding DNA methylation value of all the other cancers.

### Candidate Marker MethyLight Assay Development and Verification

We designed and tested a total of 15 real time PCR-based MethyLight assays (markers) for the ten remaining probes. MethyLight-based techniques are highly sensitive methods for detection of methylated DNA molecules [27]. The primer and probe sequences for these reactions are described in Table S1. The sequence of verification tests performed on these markers is illustrated in the right panel of Figure 1. Three MethyLight reactions did not amplify *in vitro* methylated (M.*Sss*I-treated) control DNA and were therefore eliminated. Five markers that were positive in healthy PBL samples and three markers that had a PMR<10 in more than one of 25 CRC tumors tested by MethyLight were also eliminated. The four remaining markers were tested in plasma samples from ten healthy donors and none of them were detected in these samples (data not shown). All four markers were next analyzed by Digital MethyLight in pooled serum and plasma samples from controls with or without IBD and CRC patients. Two markers that could not be detected in the pooled CRC samples were eliminated (data not shown). The final two candidate DNA methylation biomarkers that met all our stringent selection criteria were: *THBD* (THBD-M) and *C9orf50* (C9orf50-M).

### Preliminary performance evaluation of *THBD* and *C9orf50*

**Figure 3:**
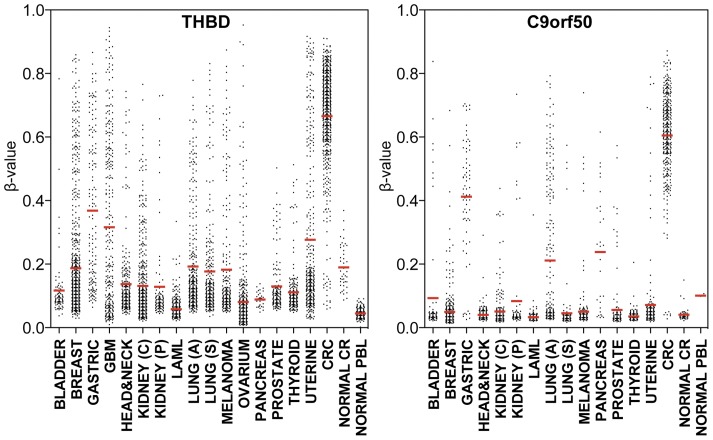
DNA methylation β-values of THBD and C9orf50 in various types of samples. Jitter plots representing Infinium-based DNA methylation β-values of *THBD* (left panel) and *C9orf50* (right panel) in 335 independent CRC tumors, matched normal colon tissues, a variety of other cancer types and PBL from healthy individuals. The specific number of samples for each tissue type is described in Table 1.

We evaluated the performance of *THBD* (Infinium probe number cg24562819) and *C9orf50* (Infinium probe number cg14015706) in discriminating CRC tissue and adjacent-normal colorectal tissue in an independent data set of 335 CRC tumors (Table 1). *THBD* and *C9orf50* had β-values >0.4 in 95% and 100% of all analyzed CRC tumors respectively. Figure 2 shows the receiver operating characteristic (ROC) curves for *THBD* and *C9orf50* in the discrimination of CRC tumor samples versus normal colonic tissue. The AUCs for *THBD* and *C9orf50* were 0.97, and 1.0, respectively. Importantly, both markers revealed lower DNA methylation levels in all other cancer types including breast, lung, prostate, thyroid, uterine, kidney, ovarian, gastric, pancreatic and bladder cancers, as well as melanoma, acute myeloid leukemia, glioblastoma multiforme and head and neck squamous cell carcinoma (Figure 3).

### Testing the performance of THBD-M and C9orf50-M in individual clinical samples

We developed a multiplex reaction for the two markers using different reporter dyes for each of the reactions. The THBD-M probe was labeled with a QUASAR fluorophore that results in a red fluorescent signal and the C9orf50-M probe was labeled with the blue FAM fluorophore. The primers and probes of the two markers were tested for interference by combining them in one solution at various concentrations using M.*Sss*I treated control DNA for MethyLight and Digital MethyLight assays (data not shown). Since the multiplex reaction of the two markers performed as well as the individual reactions we used the former for further clinical testing.

A total of 106 CRC patients and 98 controls without CRC, verified by colonoscopy, were included in this prospective study. Paired serum and plasma samples were available from all controls and 103 CRC patients, while only plasma was obtained from three CRC patients. Although stage IV CRC was an exclusion criterion in this study, aspecific abnormalities were seen on pre-operative imaging diagnostics for three patients (e.g. small pulmonary nodules on CT-thorax) which later, but before surgery, turned out to be distant metastasis. These patients were subsequently upstaged to stage IV CRC. Thirty-two plasma and serum samples from controls and CRC patients were previously used in the pooled sample analysis as mentioned above.

**Figure 4:**
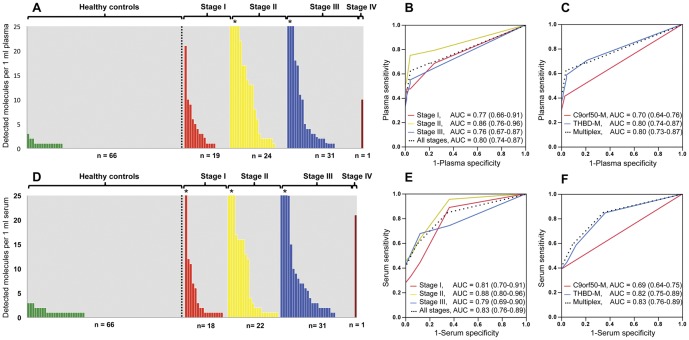
Detection of THBD-M and C9orf50-M in plasma and serum from CRC patients and controls. Digital MethyLight was performed in 1 ml plasma (A) and serum (D) to detect THBD-M and C9orf50-M in CRC and control samples. The absolute number of molecules detected by the multiplex (sum of the two markers) reaction is recorded on the y-axis. The CRC samples are arranged by stage. Asterisks (*) indicate samples with more than 25 molecules detected (up to 153 molecules in plasma and 157 molecules in serum). ROC curves and AUCs (95% confidence intervals) of the different CRC stages in plasma (B) and serum (E) based on the number of detected molecules. ROC analysis and AUCs (95% confidence intervals) for THBD-M, C9orf50-M as individual reactions and as a multiplex reaction in plasma (C) and serum (F).

We tested the multiplexed Digital MethyLight assays for THBD-M and C9orf50-M markers on individual plasma samples from 75 CRC and 66 controls and on individual serum samples from 72 CRC and 66 controls. Figure 4 shows the number of molecules (sum of the two markers) detected in 1 ml of plasma (panel A) and serum (panel D) for different stages of CRC compared to controls. The ROC curves illustrate the test performance for the multiplex reaction per disease stage (panel B) and for both markers separately in plasma (panel C) and serum (panel E and F). The AUCs per disease stage are described in Figure 4. For all stages, there was borderline significantly improved test performance for serum compared to plasma as the test medium (p = 0.06 for all stages of CRC). THBD-M performed significantly better compared to C9orf50-M in both plasma and serum (p<0.001). The addition of C9orf50-M to THBD-M for the detection of CRC did not improve test performance. The AUCs per stage, for each marker separately and the multiplex reaction, are summarized in Table S2.

We also determined the CEA levels in preoperative serum samples from 107 CRC patients. An elevated serum CEA (≥5.0 ng/ml) was observed in 35/107 (33%) patients. For stage I CRC serum CEA was elevated in 14%, for stage II in 33%, for stage III in 39% and for stage IV in 67%. Table S3 summarizes preoperative CEA serum levels of all patients with the associated number of detected THBD-M and C9orf50-M molecules per 1 ml of plasma and serum.

## Discussion

One of the important shortcomings in the published CRC biomarker studies is the reliance on a candidate gene approach for marker discovery. This approach is often based on a nonsystematic selection of candidate marker genes, which are tested in healthy and cancerous tissues and then validated in a patient population [9]–[22]. Although some of these studies have resulted in promising biomarkers for early CRC detection, the lack of a thorough biomarker discovery strategy raises the question whether superior markers may have been overlooked. With the more advanced technologies currently available, it is possible to obtain genome-scale DNA methylation data that can be useful for biomarker discovery. We performed a genome-scale multistep marker discovery to identify CRC biomarkers using the HM27 and HM450 BeadChip platform; the latter evaluates the DNA methylation status of over 482,000 CpG loci and covers 96% of all UCSC CpG islands. We recently conducted a similar marker-pipeline strategy for ovarian cancer and identified the new sensitive recurrence biomarker IFFO1-M [25]. For the present study we improved our discovery strategy by using DNA methylation data from 4,201 cancer samples of different origins to optimize CRC specificity. Our findings show that this discovery strategy works successfully for CRC, resulting in two new biomarkers: THBD-M and C9orf50-M. With AUCs of 0.97 and 1.0 respectively on the Infinium assay, these two markers have an excellent ability to distinguish between CRC tumors and matched normal colon tissue. Although DNA methylation of these genes has not yet been reported in association with CRC early detection, a recent study showed that aberrant *THBD* DNA methylation was linked to gastric cancer carcinogenesis [33], which is consistent with the slightly higher DNA methylation levels observed for this marker in gastric cancer samples compared to other types of cancer (Figure 3). Nevertheless, we found significantly higher levels of *THBD* DNA methylation in CRC than in gastric cancer (p<0.001). In this study we did not explore the biological relevance of methylated *THBD* and *C9orf50* in CRC carcinogenesis. The purpose of this study was to identify markers with high sensitivity and specificity, regardless of their function. Indeed, we anticipate that many cancer-associated epigenetic changes may represent passenger events, rather than drivers of oncogenesis.

The application of Digital PCR to multiplexed MethyLight assays allowed for efficient use of valuable samples by simultaneously analyzing more than one marker without loss of sensitivity. This technology allows for the detection of single methylated DNA molecules against a large background of unmethylated molecules, and provides a quantitative PCR test result [30].

Circulating free cancer DNA (cfDNA) has the potential to be tumor-specific and has a relatively short half-life making it suitable as biomarker [34], [35]. Although our two markers were consistently methylated in almost all CRC tumors, demonstrated excellent tissue-based discrimination between tumor and normal mucosa and tested positive in 25 clinical CRC samples using MethyLight, we were unable to detect DNA methylated at these loci in 1 ml samples of peripheral serum or plasma for some CRC cases. It is conceivable that the use of larger analyte volumes would increase sensitivity [14], but some tumors may not shed substantial amounts of tumor-derived DNA into the bloodstream. It is thought that tumor DNA enters the bloodstream by secretion or as a result of apoptosis or necrosis of cancer cells from the primary tumor or metastatic deposits [36]. While cancer patients tend to have higher cfDNA levels than healthy subjects, concentrations of cfDNA in peripheral blood may vary significantly between individuals [35]. In a prior study on Digital Methylight PCR we showed that DNA methylation marker levels in plasma of breast cancer patients are not directly related to the total yield of cfDNA as detected by an ALU reaction. For this reason we did not measure the amount of recovered cfDNA before performing Digital Methylight and chose to compare equal volumes of analyte that were processed in a standardized protocol [30].

One of the technical factors that could influence diagnostic performance of a biomarker is test volume. For example, the *SEPT9* assay utilizes 4–5 ml of plasma [15], [37]. A few studies, however, have demonstrated that *SEPT9* is also methylated in other types of cancer such as in lung adenocarcinoma [38], breast cancer [39] and head and neck squamous cell carcinoma [40], [41]. We showed that *THBD* and *C9orf50* harbor low levels of DNA methylation in 15 types of cancer other than CRC, including most high-incidence cancers. We anticipate that further assay optimization should produce substantially improved marker performance for both THBD-M and C9orf50-M.

While in this study, the use of serum resulted in a slightly higher test performance of THBD-M and the multiplex compared to plasma, this difference was of borderline significance. Although it has been reported that serum contains more cfDNA than plasma, no large-scale studies have been published comparing serum and plasma as test medium for blood-based detection of malignant diseases. Hence, it remains unclear whether serum or plasma is the optimal test specimen [36], [42].

THBD-M outperformed C9orf50-M, and combining the two markers in a multiplexed assay did not increase test sensitivity. With a detection threshold of zero molecules per 1 ml plasma, THBD-M was able to detect 71% of all CRCs at a specificity of 80%. Interestingly, for stage I/II the detection rate in CRC was 74% with this marker. THBD-M had a higher sensitivity for the detection of colon cancers (77% for all stages) than rectal tumors (53% for all stages) in plasma. This difference was marginally significant (p = 0.07). Early stage colon cancers were also detected by this marker at a relatively high percentage, 75% for stage I, and 77% for stage II. It is known that a subset of right-sided colon tumors exhibits high frequency of DNA hypermethylation at multiple promoter CpG islands, which is designated as CIMP [24], [43]. In addition it has been described that CIMP-high frequency increases gradually from the rectum to the right-sided colon [44]. We did not observe significant differences in DNA methylation of THBD-M or C9orf50-M between CIMP and non-CIMP colorectal tumors. Both markers showed high levels of DNA methylation in all colorectal cancers, regardless of their CIMP status.

The fact that this diagnostic test detected a considerable fraction of mostly curable CRCs, with 5-year survival rates of 72%–93% [45], seems promising. With an AUC of 0.80 in plasma and 0.82 in serum, THBD-M compares favorably to or outperforms other published blood-based DNA methylation biomarkers [9]–[22].

Currently, no blood-based markers have yet been approved by the FDA for the use of early detection of CRC. Serum CEA is the only blood-based biomarker that is in use for CRC detection, but it lacks the sensitivity for primary CRC detection. Serum CEA measurement is used mainly as a follow-up tool after initial treatment, and yields a sensitivity of approximately 72% for the detection of liver metastasis and 60% for local recurrence with specificities of 91% and 86% respectively [46]. In the present study, serum CEA detected only 33% of the primary CRCs.

In conclusion, we identified two novel blood-based DNA methylation markers for early detection of CRC though a systematic genome-scale marker discovery and verification study. Of these two markers, THBD-M had a promising performance in clinical samples justifying its further optimization and clinical testing.

## Supporting Information

Document S1 **Data archives used in the marker discovery study.**
(PDF)

Table S1 **MethyLight Primer and Probe Sequences.**
(PDF)

Table S2 **Area's under the curve of the biomarkers separately and combined tested in 1 ml of plasma (A) and serum (B).**
(PDF)

Table S3 **Pretherapeutical values of serum CEA and DNA methylation markers for each CRC patient.**
(PDF)
