# Supplementary material for: Genome-Scale Discovery of DNA-Methylation Biomarkers for Blood-Based Detection of Colorectal Cancer
Source: PLoS One. 2012 Nov 28;7(11):e50266. doi: 10.1371/journal.pone.0050266 (PMC3508917; doi:10.1371/journal.pone.0050266)
Supplement: Document S1 — Data archives used in the marker discovery study. (PDF) [file pone.0050266.s001.pdf]

Supplemental Document S1. Data archives used in the marker discovery study.

Datasets from Gene Expression Omnibus (GEO)
URL: http://www.ncbi.nlm.nih.gov/geo/

Infinium HumanMethylation 27 platform

Colorectal cancer HM27
Accession number GSE25062
URL: http://www.ncbi.nlm.nih.gov/geo/query/acc.cgi?acc=GSE25062

Peripheral blood lymphocytes HM27
1. Accession number GSE26989
URL: http://www.ncbi.nlm.nih.gov/geo/query/acc.cgi?acc=GSE26989
2. Accession number GSE19711
URL: http://www.ncbi.nlm.nih.gov/geo/query/acc.cgi?acc=GSE19711

Normal colorectal tissue HM27
Accession number GSE25062
URL: http://www.ncbi.nlm.nih.gov/geo/query/acc.cgi?acc=GSE26989

Datasets from The Cancer Genome Atlas (TCGA)
URL: http://tcga-data.nci.nih.gov/tcga/tcgaHome2.jsp

Infinium HumanMethylation 27 platform

Breast invasive carcinoma HM27
jhu-usc.edu_BRCA.HumanMethylation27.Level_3.1.0.0.tar
jhu-usc.edu_BRCA.HumanMethylation27.Level_3.2.0.0.tar
jhu-usc.edu_BRCA.HumanMethylation27.Level_3.3.0.0.tar
jhu-usc.edu_BRCA.HumanMethylation27.Level_3.4.0.0.tar

Colon adenocarcinoma HM27
jhu-usc.edu_COAD.HumanMethylation27.Level_3.1.2.0.tar
jhu-usc.edu_COAD.HumanMethylation27.Level_3.2.2.0.tar
jhu-usc.edu_COAD.HumanMethylation27.Level_3.3.1.0.tar
jhu-usc.edu_COAD.HumanMethylation27.Level_3.4.1.0.tar
jhu-usc.edu_COAD.HumanMethylation27.Level_3.5.2.0.tar
jhu-usc.edu_COAD.HumanMethylation27.Level_3.6.0.0.tar
jhu-usc.edu_COAD.HumanMethylation27.Level_3.7.0.0.tar
jhu-usc.edu_COAD.HumanMethylation27.Level_3.8.0.0.tar

Glioblastoma multiforme HM27
jhu-usc.edu_GBM.HumanMethylation27.Level_3.1.3.0.tar
jhu-usc.edu_GBM.HumanMethylation27.Level_3.2.2.0.tar
jhu-usc.edu_GBM.HumanMethylation27.Level_3.3.2.0.tar
jhu-usc.edu_GBM.HumanMethylation27.Level_3.4.2.0.tar

jhu-usc.edu_GBM.HumanMethylation27.Level_3.5.2.0.tar
jhu-usc.edu_GBM.HumanMethylation27.Level_3.6.0.0.tar
jhu-usc.edu_GBM.HumanMethylation27.Level_3.7.1.0.tar
jhu-usc.edu_GBM.HumanMethylation27.Level_3.8.0.0.tar
jhu-usc.edu_GBM.HumanMethylation27.Level_3.9.0.0.tar

Kidney renal clear cell carcinoma HM27
jhu-usc.edu_KIRC.HumanMethylation27.Level_3.1.2.0.tar
jhu-usc.edu_KIRC.HumanMethylation27.Level_3.2.2.0.tar
jhu-usc.edu_KIRC.HumanMethylation27.Level_3.3.2.0.tar
jhu-usc.edu_KIRC.HumanMethylation27.Level_3.4.2.0.tar
jhu-usc.edu_KIRC.HumanMethylation27.Level_3.5.2.0.tar

Kidney renal papillary cell carcinoma HM27
jhu-usc.edu_KIRP.HumanMethylation27.Level_3.1.0.0.tar

Acute Myeloid Leukemia HM27
jhu-usc.edu_LAML.HumanMethylation27.Level_3.1.4.0.tar

Lung adenocarcinoma HM27
jhu-usc.edu_LUAD.HumanMethylation27.Level_3.1.1.0.tar
jhu-usc.edu_LUAD.HumanMethylation27.Level_3.2.0.0.tar
jhu-usc.edu_LUAD.HumanMethylation27.Level_3.3.0.0.tar
jhu-usc.edu_LUAD.HumanMethylation27.Level_3.4.0.0.tar

Lung squamous cell carcinoma HM27
jhu-usc.edu_LUSC.HumanMethylation27.Level_3.1.4.0.tar
jhu-usc.edu_LUSC.HumanMethylation27.Level_3.2.4.0.tar
jhu-usc.edu_LUSC.HumanMethylation27.Level_3.3.0.0.tar
jhu-usc.edu_LUSC.HumanMethylation27.Level_3.4.0.0.tar
jhu-usc.edu_LUSC.HumanMethylation27.Level_3.5.0.0.tar

Ovarian serous cystadenocarcinoma HM27
jhu-usc.edu_OV.HumanMethylation27.Level_3.1.4.0.tar
jhu-usc.edu_OV.HumanMethylation27.Level_3.2.4.0.tar
jhu-usc.edu_OV.HumanMethylation27.Level_3.3.4.0.tar
jhu-usc.edu_OV.HumanMethylation27.Level_3.4.3.0.tar
jhu-usc.edu_OV.HumanMethylation27.Level_3.5.2.0.tar
jhu-usc.edu_OV.HumanMethylation27.Level_3.6.2.0.tar
jhu-usc.edu_OV.HumanMethylation27.Level_3.7.2.0.tar
jhu-usc.edu_OV.HumanMethylation27.Level_3.8.2.0.tar
jhu-usc.edu_OV.HumanMethylation27.Level_3.9.0.0.tar
jhu-usc.edu_OV.HumanMethylation27.Level_3.10.0.0.tar
jhu-usc.edu_OV.HumanMethylation27.Level_3.11.1.0.tar
jhu-usc.edu_OV.HumanMethylation27.Level_3.12.1.0.tar
jhu-usc.edu_OV.HumanMethylation27.Level_3.13.0.0.tar

Rectum adenocarcinoma HM27
jhu-usc.edu_READ.HumanMethylation27.Level_3.1.3.0.tar
jhu-usc.edu_READ.HumanMethylation27.Level_3.2.2.0.tar
jhu-usc.edu_READ.HumanMethylation27.Level_3.3.2.0.tar

jhu-usc.edu_READ.HumanMethylation27.Level_3.4.2.0.tar
jhu-usc.edu_READ.HumanMethylation27.Level_3.5.2.0.tar
jhu-usc.edu_READ.HumanMethylation27.Level_3.6.0.0.tar

Gastric adenocarcinoma HM27
jhu-usc.edu_STAD.HumanMethylation27.Level_3.1.0.0.tar
jhu-usc.edu_STAD.HumanMethylation27.Level_3.2.0.0.tar

Uterine Corpus Endometrioid Carcinoma HM27
jhu-usc.edu_UCEC.HumanMethylation27.Level_3.1.0.0.tar
jhu-usc.edu_UCEC.HumanMethylation27.Level_3.2.0.0.tar
jhu-usc.edu_UCEC.HumanMethylation27.Level_3.3.0.0.tar

Infinium HumanMethylation 450 platform

Bladder Urothelial Carcinoma HM450
jhu-usc.edu_BLCA.HumanMethylation450.Level_2.8.3.0.tar.gz
jhu-usc.edu_BLCA.HumanMethylation450.Level_3.1.3.0.tar.gz
jhu-usc.edu_BLCA.HumanMethylation450.Level_3.2.3.0.tar.gz
jhu-usc.edu_BLCA.HumanMethylation450.Level_3.3.3.0.tar.gz
jhu-usc.edu_BLCA.HumanMethylation450.Level_3.4.3.0.tar.gz
jhu-usc.edu_BLCA.HumanMethylation450.Level_3.5.3.0.tar.gz
jhu-usc.edu_BLCA.HumanMethylation450.Level_3.6.3.0.tar.gz
jhu-usc.edu_BLCA.HumanMethylation450.Level_3.7.3.0.tar.gz
jhu-usc.edu_BLCA.HumanMethylation450.Level_3.8.3.0.tar.gz

Breast invasive adenocarcinoma HM450
jhu-usc.edu_BRCA.HumanMethylation450.Level_3.1.1.0.tar.gz
jhu-usc.edu_BRCA.HumanMethylation450.Level_3.2.1.0.tar.gz
jhu-usc.edu_BRCA.HumanMethylation450.Level_3.3.1.0.tar.gz
jhu-usc.edu_BRCA.HumanMethylation450.Level_3.4.1.0.tar.gz
jhu-usc.edu_BRCA.HumanMethylation450.Level_3.5.1.0.tar.gz
jhu-usc.edu_BRCA.HumanMethylation450.Level_3.6.1.0.tar.gz
jhu-usc.edu_BRCA.HumanMethylation450.Level_3.7.1.0.tar.gz
jhu-usc.edu_BRCA.HumanMethylation450.Level_3.8.1.0.tar.gz
jhu-usc.edu_BRCA.HumanMethylation450.Level_3.9.1.0.tar.gz
jhu-usc.edu_BRCA.HumanMethylation450.Level_3.10.1.0.tar.gz
jhu-usc.edu_BRCA.HumanMethylation450.Level_3.11.1.0.tar.gz
jhu-usc.edu_BRCA.HumanMethylation450.Level_3.12.1.0.tar.gz
jhu-usc.edu_BRCA.HumanMethylation450.Level_3.13.1.0.tar.gz
jhu-usc.edu_BRCA.HumanMethylation450.Level_3.14.1.0.tar.gz
jhu-usc.edu_BRCA.HumanMethylation450.Level_3.15.1.0.tar.gz
jhu-usc.edu_BRCA.HumanMethylation450.Level_3.16.1.0.tar.gz

Colon adenocarcinoma HM450
jhu-usc.edu_COAD.HumanMethylation450.Level_3.1.2.0.tar.gz
jhu-usc.edu_COAD.HumanMethylation450.Level_3.2.2.0.tar.gz
jhu-usc.edu_COAD.HumanMethylation450.Level_3.3.2.0.tar.gz
jhu-usc.edu_COAD.HumanMethylation450.Level_3.4.2.0.tar.gz
jhu-usc.edu_COAD.HumanMethylation450.Level_3.5.2.0.tar.gz

jhu-usc.edu_COAD.HumanMethylation450.Level_3.6.2.0.tar.gz
jhu-usc.edu_COAD.HumanMethylation450.Level_3.7.2.0.tar.gz
jhu-usc.edu_COAD.HumanMethylation450.Level_3.8.2.0.tar.gz
jhu-usc.edu_COAD.HumanMethylation450.Level_3.9.2.0.tar.gz

Kidney renal clear cell carcinoma HM450
jhu-usc.edu_KIRC.HumanMethylation450.Level_3.1.4.0.tar.gz
jhu-usc.edu_KIRC.HumanMethylation450.Level_3.2.4.0.tar.gz
jhu-usc.edu_KIRC.HumanMethylation450.Level_3.3.4.0.tar.gz
jhu-usc.edu_KIRC.HumanMethylation450.Level_3.4.4.0.tar.gz
jhu-usc.edu_KIRC.HumanMethylation450.Level_3.5.4.0.tar.gz
jhu-usc.edu_KIRC.HumanMethylation450.Level_3.6.4.0.tar.gz

Kidney renal papillary cell carcinoma HM450
jhu-usc.edu_KIRP.HumanMethylation450.Level_3.1.0.0.tar.gz
jhu-usc.edu_KIRP.HumanMethylation450.Level_3.2.0.0.tar.gz
jhu-usc.edu_KIRP.HumanMethylation450.Level_3.3.0.0.tar.gz

Acute Myeloid Leukemia HM450
jhu-usc.edu_LAML.HumanMethylation450.Level_3.2.1.0.tar.gz

Lung adenocarcinoma HM450
jhu-usc.edu_LUAD.HumanMethylation450.Level_3.1.1.0.tar.gz
jhu-usc.edu_LUAD.HumanMethylation450.Level_3.2.1.0.tar.gz
jhu-usc.edu_LUAD.HumanMethylation450.Level_3.3.1.0.tar.gz
jhu-usc.edu_LUAD.HumanMethylation450.Level_3.4.1.0.tar.gz
jhu-usc.edu_LUAD.HumanMethylation450.Level_3.5.1.0.tar.gz
jhu-usc.edu_LUAD.HumanMethylation450.Level_3.6.1.0.tar.gz
jhu-usc.edu_LUAD.HumanMethylation450.Level_3.7.1.0.tar.gz
jhu-usc.edu_LUAD.HumanMethylation450.Level_3.8.1.0.tar.gz

Lung squamous cell carcinoma HM450
jhu-usc.edu_LUSC.HumanMethylation450.Level_3.1.2.0.tar.gz
jhu-usc.edu_LUSC.HumanMethylation450.Level_3.2.2.0.tar.gz
jhu-usc.edu_LUSC.HumanMethylation450.Level_3.3.2.0.tar.gz
jhu-usc.edu_LUSC.HumanMethylation450.Level_3.4.2.0.tar.gz
jhu-usc.edu_LUSC.HumanMethylation450.Level_3.5.2.0.tar.gz
jhu-usc.edu_LUSC.HumanMethylation450.Level_3.6.2.0.tar.gz
jhu-usc.edu_LUSC.HumanMethylation450.Level_3.7.2.0.tar.gz

Pancreatic adenocarcinoma HM450
jhu-usc.edu_PAAD.HumanMethylation450.Level_3.1.0.0.tar.gz
jhu-usc.edu_PAAD.HumanMethylation450.Level_3.2.0.0.tar.gz

Kidney renal papillary cell carcinoma HM450
jhu-usc.edu_KIRP.HumanMethylation450.Level_3.1.0.0.tar.gz
jhu-usc.edu_KIRP.HumanMethylation450.Level_3.2.0.0.tar.gz
jhu-usc.edu_KIRP.HumanMethylation450.Level_3.3.0.0.tar.gz

Prostate adenocarcinoma HM450

jhu-usc.edu_PRAD.HumanMethylation450.Level_3.1.0.0.tar.gz
jhu-usc.edu_PRAD.HumanMethylation450.Level_3.2.0.0.tar.gz
jhu-usc.edu_PRAD.HumanMethylation450.Level_3.3.0.0.tar.gz
jhu-usc.edu_PRAD.HumanMethylation450.Level_3.4.0.0.tar.gz

Rectum adenocarcinoma HM450
jhu-usc.edu_READ.HumanMethylation450.Level_3.1.0.0.tar.gz
jhu-usc.edu_READ.HumanMethylation450.Level_3.2.0.0.tar.gz
jhu-usc.edu_READ.HumanMethylation450.Level_3.3.0.0.tar.gz
jhu-usc.edu_READ.HumanMethylation450.Level_3.4.0.0.tar.gz
jhu-usc.edu_READ.HumanMethylation450.Level_3.5.0.0.tar.gz
jhu-usc.edu_READ.HumanMethylation450.Level_3.6.0.0.tar.gz

Skin Cutaneous Melanoma HM450
jhu-usc.edu_SKCM.HumanMethylation450.Level_3.1.0.0.tar.gz
jhu-usc.edu_SKCM.HumanMethylation450.Level_3.2.0.0.tar.gz

Gastric adenocarcinoma HM450
jhu-usc.edu_STAD.HumanMethylation450.Level_3.1.1.0.tar.gz
jhu-usc.edu_STAD.HumanMethylation450.Level_3.2.1.0.tar.gz
jhu-usc.edu_STAD.HumanMethylation450.Level_3.3.1.0.tar.gz

Thyroid carcinoma HM450
jhu-usc.edu_THCA.HumanMethylation450.Level_3.1.0.0.tar.gz
jhu-usc.edu_THCA.HumanMethylation450.Level_3.2.0.0.tar.gz
jhu-usc.edu_THCA.HumanMethylation450.Level_3.3.0.0.tar.gz
jhu-usc.edu_THCA.HumanMethylation450.Level_3.4.0.0.tar.gz
jhu-usc.edu_THCA.HumanMethylation450.Level_3.5.0.0.tar.gz
jhu-usc.edu_THCA.HumanMethylation450.Level_3.6.0.0.tar.gz
jhu-usc.edu_THCA.HumanMethylation450.Level_3.7.0.0.tar.gz
jhu-usc.edu_THCA.HumanMethylation450.Level_3.8.0.0.tar.gz
jhu-usc.edu_THCA.HumanMethylation450.Level_3.9.0.0.tar.gz

Uterine Corpus Endometrioid Carcinoma HM450
jhu-usc.edu_UCEC.HumanMethylation450.Level_3.1.0.0.tar.gz
jhu-usc.edu_UCEC.HumanMethylation450.Level_3.2.0.0.tar.gz
jhu-usc.edu_UCEC.HumanMethylation450.Level_3.3.0.0.tar.gz
jhu-usc.edu_UCEC.HumanMethylation450.Level_3.4.0.0.tar.gz
jhu-usc.edu_UCEC.HumanMethylation450.Level_3.5.0.0.tar.gz
jhu-usc.edu_UCEC.HumanMethylation450.Level_3.6.0.0.tar.gz
jhu-usc.edu_UCEC.HumanMethylation450.Level_3.7.0.0.tar.gz
jhu-usc.edu_UCEC.HumanMethylation450.Level_3.8.0.0.tar.gz
jhu-usc.edu_UCEC.HumanMethylation450.Level_3.9.0.0.tar.gz
jhu-usc.edu_UCEC.HumanMethylation450.Level_3.10.0.0.tar.gz
jhu-usc.edu_UCEC.HumanMethylation450.Level_3.11.0.0.tar.gz
jhu-usc.edu_UCEC.HumanMethylation450.Level_3.12.0.0.tar.gz
