# Supplementary material for: Genome-Scale Discovery of DNA-Methylation Biomarkers for Blood-Based Detection of Colorectal Cancer
Source: PLoS One. 2012 Nov 28;7(11):e50266. doi: 10.1371/journal.pone.0050266 (PMC3508917; doi:10.1371/journal.pone.0050266)
Supplement: Table S1 — MethyLight Primer and Probe Sequences. (PDF) [file pone.0050266.s002.pdf]

Supplemental Table S1. MethyLight Primer and Probe Sequences.

Gene	MethyLight reaction ID number	Forward Primer Sequence	Reverse Primer Sequence	Probe Sequence
TFPI2	HB-628	GTTAAATTCGCGTATGATTTCGAGA	TTCCCGCGTCTCCGAC	6FAM-AACGAACGACCCTCTCGCTCCGAA-BHQ-1
THBD	HB-971	GTTGTTTTTCGTAATTTATTGGAAGTC	GACCTACAACTACCACCCGACTA	QUASAR670-CGACGACCCCAAACGCCTCG-BHQ3
C9orf50	HB-930	GAGTTTTTTGGGCGTTAGGT	CGCTAAAAATACGATAAACTAAATACG	6FAM-TCTAACGTCGACTTCGCCCAAAAACC-BHQ1
C9orf50	HB-932	TTTTTAGGAAGGCGTTTAAGAAGTC	GTCCCGAAAATCGACGC	6FAM-GAACGCCCCCGAAAACGCGTA-BHQ1
C9orf50	HB-933	TTTTTAGGAAGGCGTTTAAGAAGTC	CGAAAATCGACGCCCA	6FAM-GAACGCCCCCGAAAACGCGTA-BHQ1
ADHFE1	HB-934	TAGATAGGTGATTTCGCGAAGC	CGCCGACCAATCACG	6FAM-CCTCGACCGCGCCTACCCAC-BHQ1
ADHFE1	HB-935	TAGATAGGTGATTTCGCGAAGC	TCCCTCCTCGAATCGCTA	6FAM-GAACGCCCCCGAAAACGCGTA-BHQ1
FGF12	HB-936	TCGCGCGTTTCGGTA	ACTTCCTTCCTCGACCGAA	6FAM-AATCGACCTTACGTCCGACGAATAATCCC-BHQ1
PTPRT	HB-937	GTCGGGTAGTGGCGGTAGT	CGCCCGAATAACGAACC	6FAM-CCGCGCTCGCCCTCAACCTA-BHQ1
ZNF568	HB-938	TCGGCGTTTTAGGCGTT	ACCTACCGAAAACACCCGA	6FAM-AAAACGACCCGCAAATA-MGBFNQ
KIAA1026	HB-939	TTTATATTCGGGGCGAAATC	AACCTACAACTCCCAACCGA	6FAM-ACAACGATAACCACCGCCGAACG-BHQ1
SFMBT2	HB-940	GTTGGGTTTTTAATGGTTTAGTCG	TTATCTCCGCTCCCAACG	6FAM-CGAATAAAACGCAAATTTATAC-MGBFNQ
SFMBT2	HB-941	AAAACGTTTTTAGGTATTTGGTCG	ACGATCCCCGAAACTAACG	6FAM-CTCCTCCGAACCCGCGAACCTAATC-BHQ1
AUTS2	HB-942	AGGAAGATAATGGGAAGTTTTCGT	TTCTACCGAAATAAACTCTCTCCG	6FAM-CCGCGAAAATCTAAACC-MGBFNQ
C9orf50	HB-948	TTTTTTAGGAAGGCGTTTAAGAAGT	GACGCGTCCCGAAAATC	6FAM-AAAACGCGAACGCCCCCGA-BHQ1

The primers and probes are written in the 5' to 3' orientation. All probes a 6FAM fluorophore at the 5' end except for THBD which contains a QUASAR670 fluorophore.
All probes have a Black Hole Quencher
