# Supplementary material for: Genome-Scale Discovery of DNA-Methylation Biomarkers for Blood-Based Detection of Colorectal Cancer
Source: PLoS One. 2012 Nov 28;7(11):e50266. doi: 10.1371/journal.pone.0050266 (PMC3508917; doi:10.1371/journal.pone.0050266)
Supplement: Table S2 — Area's under the curve of the biomarkers separately and combined tested in 1 ml of plasma (A) and serum (B). (PDF) [file pone.0050266.s003.pdf]

Supplemental Table S2. AUCs of the biomarkers separately and combined tested in 1 ml of plasma (A) and serum (B).

A

**Marker**	**Stage 1 (95% CI)**	**Stage 2 (95% CI)**	**Stage 3 (95% CI)**	**All stages (95% CI)**
THBD-M	0.78 (0.66-0.91)	0.86 (0.76-0.96)	0.77 (0.67-0.87)	0.80 (0.74-0.87)
C9orf50-M	0.65 (0.53-0.76)	0.78 (0.68-0.89)	0.65 (0.56-0.74)	0.70 (0.64-0.76)
Multiplex	0.78 (0.65-0.90)	0.86 (0.76-0.96)	0.76 (0.66-0.87)	0.80 (0.73-0.87)

B

**Marker**	**Stage 1 (95% CI)**	**Stage 2 (95% CI)**	**Stage 3 (95% CI)**	**All stages (95% CI)**
THBD-M	0.80 (0.69-0.91)	0.88 (0.80-0.95)	0.79 (0.68-0.89)	0.82 (0.75-0.88)
C9orf50-M	0.64 (0.53-0.75)	0.75 (0.64-0.86)	0.69 (0.61-0.78)	0.69 (0.64-0.75)
Multiplex	0.81 (0.70-0.91)	0.88 (0.80-0.96)	0.79 (0.69-0.90)	0.83 (0.76-0.89)
