# Supplementary material for: Genome-Scale Discovery of DNA-Methylation Biomarkers for Blood-Based Detection of Colorectal Cancer
Source: PLoS One. 2012 Nov 28;7(11):e50266. doi: 10.1371/journal.pone.0050266 (PMC3508917; doi:10.1371/journal.pone.0050266)
Supplement: Table S3 — Pretherapeutical values of serum CEA and DNA methylation markers for each CRC patient. (PDF) [file pone.0050266.s004.pdf]

Supplemental Table S3. Pretherapeutical values of serum CEA and DNA methylation markers for each CRC patient.

Case number	Disease Stage	THBD-M Serum (1ml) Molecules per ml	THBD-M Plasma (1ml) Molecules per ml	C9orf50-M Serum (1ml) Molecules per ml	C9orf50-M Plasma (1ml) Molecules per ml	CEA Serum ng/ml
1	I	1	3	0	0	0.7
2	I	7	5	0	0	0.7
3	I	N/A	N/A	N/A	N/A	1.4
4	I	N/A	N/A	N/A	N/A	1.5
5	I	5	8	1	2	1.6
6	I	8	4	3	2	1.8
7	I	1	1	0	0	1.8
8	I	1	0	0	0	1.9
9	I	1	0	0	0	1.9
10	I	1	2	0	0	2.0
11	I	N/A	N/A	N/A	N/A	2.2
12	I	N/A	N/A	N/A	N/A	2.2
13	I	1	1	0	0	2.4
14	I	3	0	0	0	2.6
15	I	N/A	N/A	N/A	N/A	2.6
16	I	N/A	N/A	N/A	N/A	2.6
17	I	27	15	6	6	2.7
18	I	1	1	0	0	2.7
19	I	1	3	0	1	2.7
20	I	N/A	0	N/A	0	2.8
21	I	N/A	N/A	N/A	N/A	3.2
22	I	10	8	2	1	3.9
23	I	0	0	0	0	4.2
24	I	N/A	N/A	N/A	N/A	4.2
25	I	1	0	1	0	7.4
26	I	2	1	0	0	9.1
27	I	N/A	N/A	N/A	N/A	10.3
28	I	0	2	0	1	28.0
29	II	63	11	30	18	0.5
30	II	N/A	1	N/A	0	1.0
31	II	8	15	8	17	1.1
32	II	N/A	N/A	N/A	N/A	1.5
33	II	N/A	N/A	N/A	N/A	1.6
34	II	N/A	N/A	N/A	N/A	1.6
35	II	15	11	2	2	1.7
36	II	2	2	0	0	1.7
37	II	80	7	18	22	2.3
38	II	1	2	0	1	2.3
39	II	8	10	5	4	2.5
40	II	1	6	0	2	3.2
41	II	2	2	0	0	3.4
42	II	1	0	0	0	3.5
43	II	N/A	N/A	N/A	N/A	3.8
44	II	N/A	N/A	N/A	N/A	3.9
45	II	1	0	0	0	4.4
46	II	1	2	0	0	4.4
47	II	0	1	0	1	4.5
48	II	N/A	N/A	N/A	N/A	5.0
49	II	1	2	0	0	5.6

50	II	2	0	0	0	6.2
51	II	12	10	4	3	8.1
52	II	95	90	21	39	12.7
53	II	7	10	5	7	13.1
54	II	2	0	1	0	14.5
55	II	13	15	3	2	22.0
56	II	1	0	0	0	24.1
57	II	3	5	1	1	31.0
58	II	N/A	19	N/A	3	70.4
59	III	0	0	0	0	1.0
60	III	0	0	0	0	1.0
61	III	7	3	0	0	1.1
62	III	2	0	0	0	1.1
63	III	1	1	0	0	1.2
64	III	4	0	3	0	1.2
65	III	N/A	N/A	N/A	N/A	1.3
66	III	2	4	1	0	1.6
67	III	0	0	0	0	1.8
68	III	0	2	0	0	1.9
69	III	N/A	N/A	N/A	N/A	1.9
70	III	10	10	5	7	2.0
71	III	1	0	0	0	2.0
72	III	N/A	N/A	N/A	N/A	2.0
73	III	4	0	0	0	2.1
74	III	3	1	0	0	2.2
75	III	2	2	0	1	2.3
76	III	N/A	N/A	N/A	N/A	2.5
77	III	N/A	N/A	N/A	N/A	2.5
78	III	N/A	2	N/A	0	2.6
79	III	4	N/A	1	N/A	2.6
80	III	0	0	0	0	3.1
81	III	0	0	0	0	3.3
82	III	93	96	64	54	3.4
83	III	0	0	0	0	3.6
84	III	N/A	N/A	N/A	N/A	3.9
85	III	3	3	0	1	4.5
86	III	21	16	4	2	4.8
87	III	N/A	N/A	N/A	N/A	5.5
88	III	0	0	0	0	6.1
89	III	N/A	N/A	N/A	N/A	6.6
90	III	9	5	1	0	7.4
91	III	N/A	N/A	N/A	N/A	8.9
92	III	2	2	0	0	9.1
93	III	2	1	0	0	11.0
94	III	6	8	0	3	11.9
95	III	4	8	4	2	18.3
96	III	6	1	3	0	18.7
97	III	N/A	N/A	N/A	N/A	20.7
98	III	N/A	N/A	N/A	N/A	21.0
99	III	N/A	N/A	N/A	N/A	22.1
100	III	N/A	N/A	N/A	N/A	23.9
101	III	N/A	N/A	N/A	N/A	32.4
102	III	80	85	14	50	35.7
103	III	N/A	1	N/A	1	54.1
104	III	94	91	59	62	78.7
105	III	1	N/A	1	N/A	N/A
106	IV	21	9	0	1	1.8
107	IV	N/A	N/A	N/A	N/A	16.0

108	IV	N/A	N/A	N/A	N/A	157.0

N/A, data not available
CEA, carcinoembryonic antigen
